# A clinical evaluation study of cardiothoracic ratio measurement using artificial intelligence

**DOI:** 10.1186/s12880-022-00767-9

**Published:** 2022-03-16

**Authors:** Pairash Saiviroonporn, Suwimon Wonglaksanapimon, Warasinee Chaisangmongkon, Isarun Chamveha, Pakorn Yodprom, Krittachat Butnian, Thanogchai Siriapisith, Trongtum Tongdee

**Affiliations:** 1 Department of Radiology, Faculty of Medicine Siriraj Hospital, Mahidol University, 2 Wanglang Road, Bangkoknoi, Bangkok, 10700 Thailand; 2 Institute of Field Robotics, King Mongkut’s University of Technology Thonburi, Bangkok, Thailand; 3 Perceptra Co., Ltd., Bangkok, Thailand

**Keywords:** Cardiothoracic ratio, Deep learning, Clinical evaluation, CXR, AI

## Abstract

**Background:**

Artificial intelligence, particularly the deep learning (DL) model, can provide reliable results for automated cardiothoracic ratio (CTR) measurement on chest X-ray (CXR) images. In everyday clinical use, however, this technology is usually implemented in a non-automated (AI-assisted) capacity because it still requires approval from radiologists. We investigated the performance and efficiency of our recently proposed models for the AI-assisted method intended for clinical practice.

**Methods:**

We validated four proposed DL models (AlbuNet, SegNet, VGG-11, and VGG-16) to find the best model for clinical implementation using a dataset of 7517 CXR images from manual operations. These models were investigated in single-model and combined-model modes to find the model with the highest percentage of results where the user could accept the results without further interaction (excellent grade), and with measurement variation within ± 1.8% of the human-operating range. The best model from the validation study was then tested on an evaluation dataset of 9386 CXR images using the AI-assisted method with two radiologists to measure the yield of excellent grade results, observer variation, and operating time. A Bland–Altman plot with coefficient of variation (CV) was employed to evaluate agreement between measurements.

**Results:**

The VGG-16 gave the highest excellent grade result (68.9%) of any single-model mode with a CV comparable to manual operation (2.12% vs 2.13%). No DL model produced a failure-grade result. The combined-model mode of AlbuNet + VGG-11 model yielded excellent grades in 82.7% of images and a CV of 1.36%. Using the evaluation dataset, the AlbuNet + VGG-11 model produced excellent grade results in 77.8% of images, a CV of 1.55%, and reduced CTR measurement time by almost ten-fold (1.07 ± 2.62 s vs 10.6 ± 1.5 s) compared with manual operation.

**Conclusion:**

Due to its excellent accuracy and speed, the AlbuNet + VGG-11 model could be clinically implemented to assist radiologists with CTR measurement.

## Introduction

**Fig. 1:**
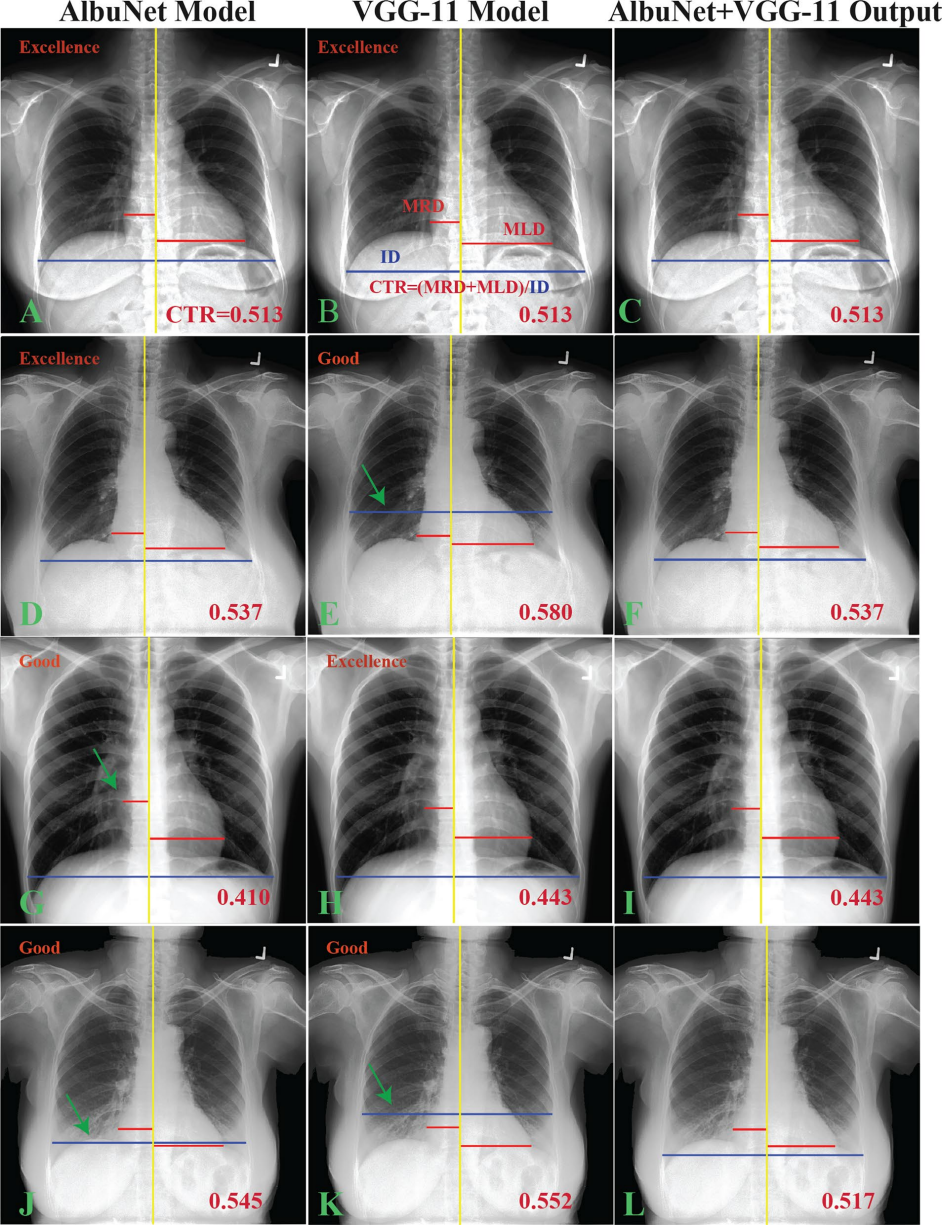
CTR measurements using AlbuNet and VGG-11 models (the first and second column) and results of the combined-model (AlbuNet + VGG-11) mode (the third column). The first (**a**–**c**)–third (**g**–**i**) rows represent examples of the excellent grade while the last row (**j**–**l**) is a good grade result. In the first row, outcomes A and B were excellent. Measurements D and H were excellent on the second and third rows, respectively. The arrows point to the error of AI calculation

Chest radiography (CXR) imaging is the most common screening modality for cardiomegaly [1–4], which is defined as the ratio of heart to internal thoracic diameters, referred to as the Cardiothoracic Ratio (CTR), (Fig. 1b). Cardiomegaly, or enlarged heart, should be suggested if the CTR value is greater than 0.5 [1], but CTR measurement is typically performed manually and is a burden to radiologists, especially if all normal and cardiomegaly cases must be measured. To ease the burden, Deep Learning (DL), a subset of Artificial Intelligence (AI), has been implemented for CTR calculation [5–11]. The AI method had been technically [6–8] and clinically [9, 10] validated for CTR measurement and can provide a reliable result with measurement variation within the human-operating range [10]. Such reliability made the automated calculation of the CTR feasible, but in actual clinical practice automated measurement has not been employed [9] because the measurements still required approval from radiologists.

In the AI-assisted method, the user is presented with the AI’s results and can choose to accept them without further adjustment, or disagree and changes as required. The preferred result is when the user can accept the AI results without further interaction, which is considered an excellent grade result in our study. In our 2021 study [9] of the AI-assisted method, we found that our model could achieve an excellent grade in only about 40% of images, lower than our desired result of around 70%. In a more recent study [10], we developed an improved model architecture and better training methodology that achieved CTR measurement with an average error on-par with manual measurement by experts. The study concluded that the improved AlbuNet model could be reliably employed for the automated calculation of CTR values.

Here, we further investigated the efficiency and reliability of all models from our recent study [10] using the AI-assisted method, and aimed to find the best model for clinical use. We performed a validation study on the models using our previous dataset [9] with manual calculation of the CTR measurement as the reference, and compared the performance of these models to find the best option for clinical implementation (i.e., the model that provided the highest proportion of excellent grade results). We then evaluated the selected model on evaluation dataset for clinical use to determine the model’s efficiency to assist radiologists to measure the CTR on all normal and cardiomegaly cases.

## Materials and methods

### Study population

This study was approved by the Siriraj Institutional Review Board (Si469/2021) and complied with the Declaration of Helsinki. Due to the retrospective nature of the study, informed consent was not required. The validation dataset was from our previous investigation (Si069/2020) of observer and method validation [9], and was employed here to compare the performance of our improved DL models to the previous one. Briefly, there were 7517 PA-upright-CXR images acquired between 2010 and 2019 from patients >17 years of age, from randomly selected normal images (5000) and all cardiomegaly images with CTR measurement reports (2517).

The evaluation dataset was utilized to determine the performance of our selected model from the validation study on clinical use. The dataset was acquired from the Picture Archiving Communication System (PACS) in our radiology department by selecting all PA-upright-CXR images with patients >17 years of age in a two-month period (1-January-2020 to 28-Feburay-2020). The dataset represented a sample of a clinical dataset required to perform CTR measurements on all patients, which differs from our current clinical setting in that our radiologists only measure CTR on suspected cardiomegaly cases. Using this dataset, we should be able to determine the performance and efficiency of our improved models using the AI-assisted method on all patients in order to determine if it should be implemented in the clinical setting. This dataset is private but is available on reasonable request.

### AI model

**Fig. 2:**
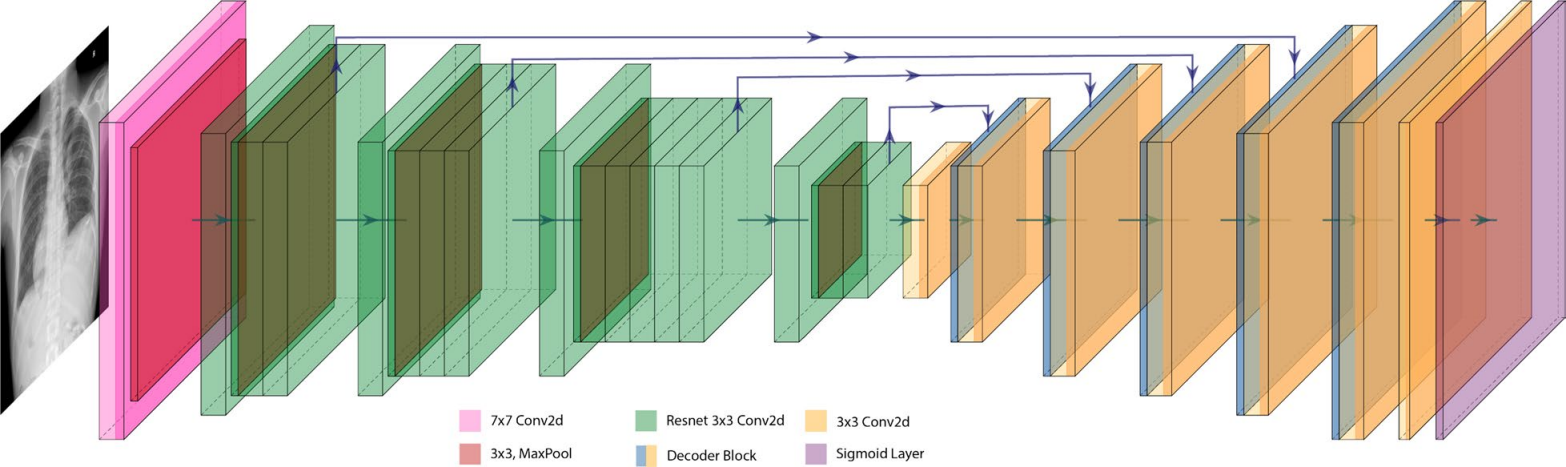
Model architecture of AlbuNet model

In our recent study [10], we reviewed the literature regarding anatomical segmentation in chest x-rays and observed that U-Net has emerged as a widely used model for chest x-ray and medical image segmentation tasks [12, 13]. As the name suggested, the U-shape architecture consists of (1) an encoder that extracts features through successive convolutional layers that reduce the dimension of the inputs, and (2) a decoder that applies successive up-sampling operators to predict a high-resolution mask output. This characteristic allows U-Net to be versatile as it can be adapted with various types of encoders and outperforms most commonly used segmentation models in the medical image domain. Hence, we focused on U-Net architecture and implemented four variants of U-Net architectures (VGG-11 U-Net, VGG-16 U-Net, SegNet, and AlbuNet) to predict the cardiac and thoracic outlines from CXR images. We customized U-Net to use the VGG network as an encoder similar to TernausNet [14], and experimented with both VGG-11 and VGG-16 variants. Furthermore, we implemented a similar architecture called SegNet [15], which utilized VGG-16 [16] architecture as an encoder and improved the decoder by reusing memorized max-pooling indices from the corresponding encoder layers in the up-sampling process. These U-Net variants showed excellent performance in biomedical image segmentations with similar challenges as chest x-ray diagnosis. Lastly, we implemented AlbuNet [17], which deploys ResNet as an encoder. The architecture of our customized AlbuNet is demonstrated in Fig. 2. All networks were pre-trained with ImageNet [18] and fined-tuned on an image repository of 485 images with lung boundary annotations and 461 images with heart boundary annotations. These images are derived from the JSRT dataset [19], Montgomery County dataset [20], ChestX-ray14 dataset [21], and the CheXpert dataset [22]. Our loss function is a sum of the Soft Dice loss and the binary cross entropy with logits loss. We trained each model using the Adam (Adaptive Moment Estimation) optimizer with a batch size of eight for 75 epochs and an initial learning rate of 0.0001. The training algorithm was implemented on an Nvidia Tesla V100 GPU with 32~GB memory.

In comparison with the model used in our previous study [9], this model set was vastly improved by (1) adding new model architecture and performing hyper-parameter optimization, (2) expanding our segmented training dataset, and (3) expanding our image augmentation repertoire to improve generalizability.

### Experimental setting

First, we validated the proposed DL models [10] to find the best model results for clinical implementation, and then evaluated the best model for clinical use. To validate the DL models, we performed the experiment on our previous dataset with manual results that served as the reference and employed the models using the AI-assisted method [9], and calculated percentage difference of CTR values between AI’s and manual results, or CTR_diff_. In short, the AI-assisted method presents the AI’s results to the user and the user can choose to accept them without further adjustment, or disagree and make the required changes. If two users independently accepted the AI’s results without adjustment, then the AI’s result was given an excellent grade. A grade of “good” was assigned if any adjustment was required. An AI failure was defined as a poor grade that required manual operation from the user.

In our previous study [9], we found that the excellent grade had CTR_diff_ in ± 1.8% range. We, thus, used this range to determine the excellent grade for our proposed DL model results and any differences greater than this range were graded as good, except for AI failure. This setup, then, can be utilized to analyze AI results without additional operations from the user. Using this approach, we aimed to find the model that provided the highest excellent grade results and then to evaluate it in a clinical setting.

Four models were validated as single-models and six models were validated in the combined-model modes (Table 1). In the single-model mode, the excellent grade was obtained from CTR_diff_ that were within the excellent range as already described, and we selected from the lowest CTR_diff_ of two models in the combination mode. The reliabilities of the proposed models were investigated. Method variations between models and manual operation were analyzed and compared to the inter-observer variation to gauge the reliability of the models. For practical purposes, the proposed models’ results should have variation compared to manual operation not more than from the inter-observer variation (i.e., the models’ results should be within the user-operative variation).

**Table 1:** AI outcomes from single and combination of two models on validation dataset

	CTR	CTR_diff_ (%)
*Single-model mode*		
AlbuNet	0.489 ± 0.074	− 0.69 ± 2.64
SegNet	0.491 ± 0.071	− 0.22 ± 3.17
VGG-11	0.502 ± 0.075	1.96 ± 3.20
VGG-16	0.494 ± 0.073	0.48 ± 2.91
*Combined-model mode*		
AlbuNet + SegNet	0.494 ± 0.072	0.39 ± 1.99
AlbuNet + VGG-11	**0.491 ± 0.071**	− **0.18 ± 1.92**
AlbuNet + VGG-16	**0.492 ± 0.072**	− **0.01 ± 1.98**
SegNet + VGG-11	**0.495 ± 0.071**	**0.63 ± 2.10**
SegNet + VGG-16	0.493 ± 0.072	0.20 ± 2.09
VGG-11 + VGG-16	0.496 ± 0.073	0.83 ± 2.38

The bold-data rows indicate CTR values of combination-model modes that were significantly different (*P* < 0.01) from each individual model before the combination

To evaluate the best model result from the validation study, we investigated intra- and inter-observer variations of CTR measurement using the AI-Assisted method on the evaluation dataset to determine the yield of excellent grade results. This dataset served as a testing dataset and was not part of the training or validation process of the models. Two thoracic-imaging radiologists (SW and KB), with 12 and 5 years of experience respectively, separately performed CTR measurement using the AI-assisted method. SW performed the measurement twice (intra-observer) and KB only once (inter-observer). The intra-observer study was performed separately and two weeks apart on each dataset to reduce measurement bias.

Our MATLAB program (R2020a, MathWorks, Inc., Natick, MA, USA) was used in the evaluation study. In short, the software provides a graphical user interface for CTR measurement and records the user-interaction time of each measurement. In the combined-model mode, users were presented with the AI’s results from two models, one of which could be selected as the desired result. If they were not satisfied with either result, then manual adjustment of the CTR measurement was performed. The results were graded as excellent when both users independently accepted the AI’s results without any adjustment, as good if any adjustment was needed, and poor if the AI failed to segment the lung or heart region. The operating time of each case was measured from the start of line adjustment to acceptance.

### Statistical analysis

Statistical analysis was performed using the MATLAB program. The paired Student’s t-test was employed for parametric evaluation of CTR_diff_ on both single-model and combined-model modes with the significance level set at *P* < 0.05. Bland–Altman plot was employed to evaluate agreement between measurement methods. Coefficient of variation (CV) signifying the level of agreement was calculated from the standard deviation of the differences between two measurements then divided by their mean and expressed as a percentage. Thus, the lower the CV the better the agreement was between two measurement methods.

## Results

### Patient characteristics

The evaluation dataset included 9755 patients but CTR could not be measured in 369 cases (3.7%) by radiologists due to the absence of demonstrable cardiac borders from pleural effusion, lung atelectasis, and mediastinal mass. Furthermore, some patients with severe thoracolumbar scoliosis could not be measured due to a severely abnormal axis and so the unmeasurable CTRs were excluded from the study. Therefore, there were total of 5685 (2143 males and 3542 females; aged 49.1 ± 17.7 years) patients with normal CXR images, and 3701 (1130 males and 2571 females; aged 64.7 ± 14.4 years) CXR images for patients with cardiomegaly as defined by a CTR value greater than 0.5 (Table 2).

**Table 2:** Patient demographic data of evaluation study

	Normal group	Cardiomegaly group
Number of patients	5685	3701
*Gender*		
Male	2143 (37.7%)	1130 (30.5%)
Female	3542 (62.3%)	2571 (69.5%)
Mean age (years)	49.1 ± 17.7	64.7 ± 14.4
*Age*		
< 18	55 (0.9%)	4 (0.1%)
18–35	1,471 (25.9%)	133 (3.6%)
36–50	1301 (22.9%)	422 (11.4%)
51–65	1748 (30.7%)	1253 (33.8%)
66–80	947 (16.7%)	1373 (37.1%)
> 80	163 (2.9%)	516 (14.0%)
CTR value	0.452 ± 0.032	0.549 ± 0.043

### AI outcomes

#### The validation study

There were no AI failure results in any of the proposed models, leaving only results graded as excellent and good. The CTR and CTR_diff_ of both single-model and combined-model modes are presented in Table 1. The CTR of all single-models were significantly different (*P* < 0.01). Only the AlbuNet+VGG-11, AlbuNet+VGG-16, and Segnet+VGG-11 provided CTR values that were significantly different (*P* < 0.01) from each individual model before the combination.

**Fig. 3:**
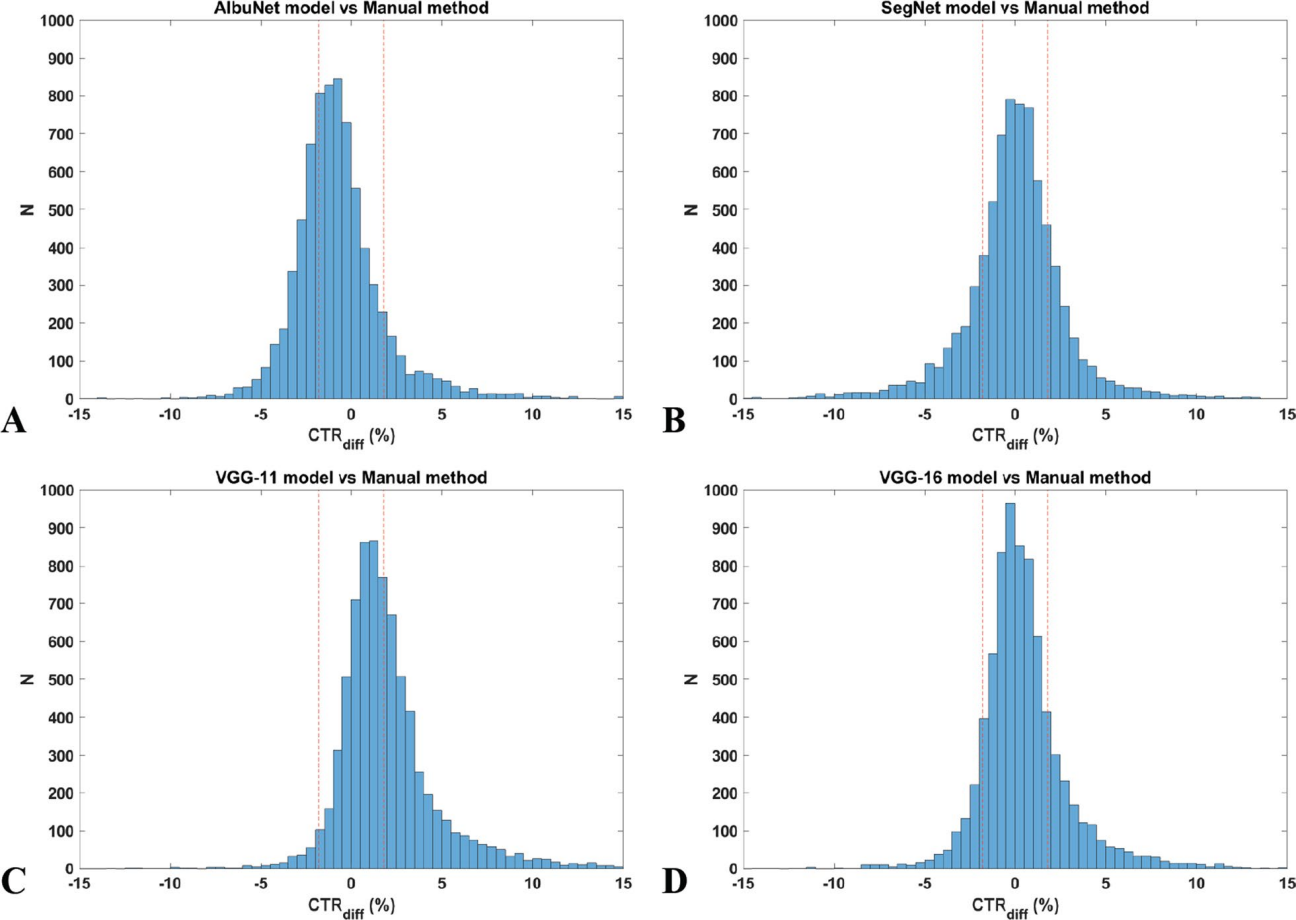
Histograms of all single-model mode with the excellent grade defined as a region between red-dashed lines (CTR_diff_ at ± 1.8%). *Note*: the CTR_diff_ from AlbuNet model was skew to the left while was to the right by VGG-11

The histograms of the CTR_diff_ of all models in the single-model mode with the excellent range defined as a region between red-dashed lines is presented in Fig. 3. The VGG-16 model had the highest population inside this range (68.9%) (Table 3), while the lowest was from VGG-11 (52.8%). The VGG-16 model, therefore, should be the best model for clinical use if the single-model mode were employed in the AI-assisted method. An interesting point in these histograms was that the CTR_diff_ from the AlbuNet model was skew to the left while the VGG-11 skewed to the right. This suggests that the AlbuNet model tends to under-estimated CTR values as compared to the manual operation, while the opposite occurred with the VGG-11. The other two models, however, had symmetric profiles.

**Table 3:** Grading of AI outcomes from single and combination of two models on validation dataset

	Excellent grade	Good grade
*Single-model mode*		
AlbuNet	4295 (57.1%)	3222 (42.9%)
SegNet	4655 (61.9%)	2862 (38.1%)
VGG-11	3971 (52.8%)	3546 (47.2%)
VGG-16	**5183 (68.9%)**	**2334 (31.1%)**
*Combined-model mode*		
AlbuNet + VGG-11	**6220 (82.7%)**	**1297 (17.3%)**
AlbuNet + VGG-16	6121 (81.4%)	1396 (18.6%)
SegNet + VGG-11	5664 (75.3%)	1853 (24.7%)

Bold values indicate the best excellent grade on each mode

The combined-model mode further improved the yield of excellent grade results. The AlbuNet+VGG-11 produced 83% excellent grade results, more than 10% higher than the VGG-16 single-model result (Table 3). Furthermore, the combined-model mode also reduced measurement variation compared to manual operation (Table 4). For example, if the single-model mode were employed, then the AlbuNet model should provide the lowest variation (CV=1.92), while the variation would be reduced to 1.36, if AlbuNet+VGG-11 were used. Thus, the combined-model mode can improve the yield of excellent grade results and reduce measurement variation. The AlbuNet+VGG-11 model, then, was selected for the evaluation study because it provides the highest return of excellent-grade results with the lowest measurement variations of all the combination models.

**Table 4:** Comparison of Bias, 95% CI, and coefficient of variation of CTR measurements from both single and combination models to manual operation on validation dataset

	Bias (95% CI) (%)	CV (%)
*Single-model mode*		
AlbuNet	− **0.69 (**− **5.86 4.48)**	**1.92**
SegNet	− 0.22 (− 6.24 6.20)	2.28
VGG-11	1.96 (− 4.32 8.23)	2.65
VGG-16	0.48 (− 5.24 6.20)	2.12
*Combined-model mode*		
Albunet + VGG-11	− **0.18 (**− **3.93 3.56)**	**1.36**
Albunet + VGG-16	− 0.01 (− 3.88 3.87)	1.39
Segnet + VGG-11	0.63 (− 3.49 4.76)	1.55

Bold values indicate the best CV on each mode

### The evaluation study

**Fig. 4:**
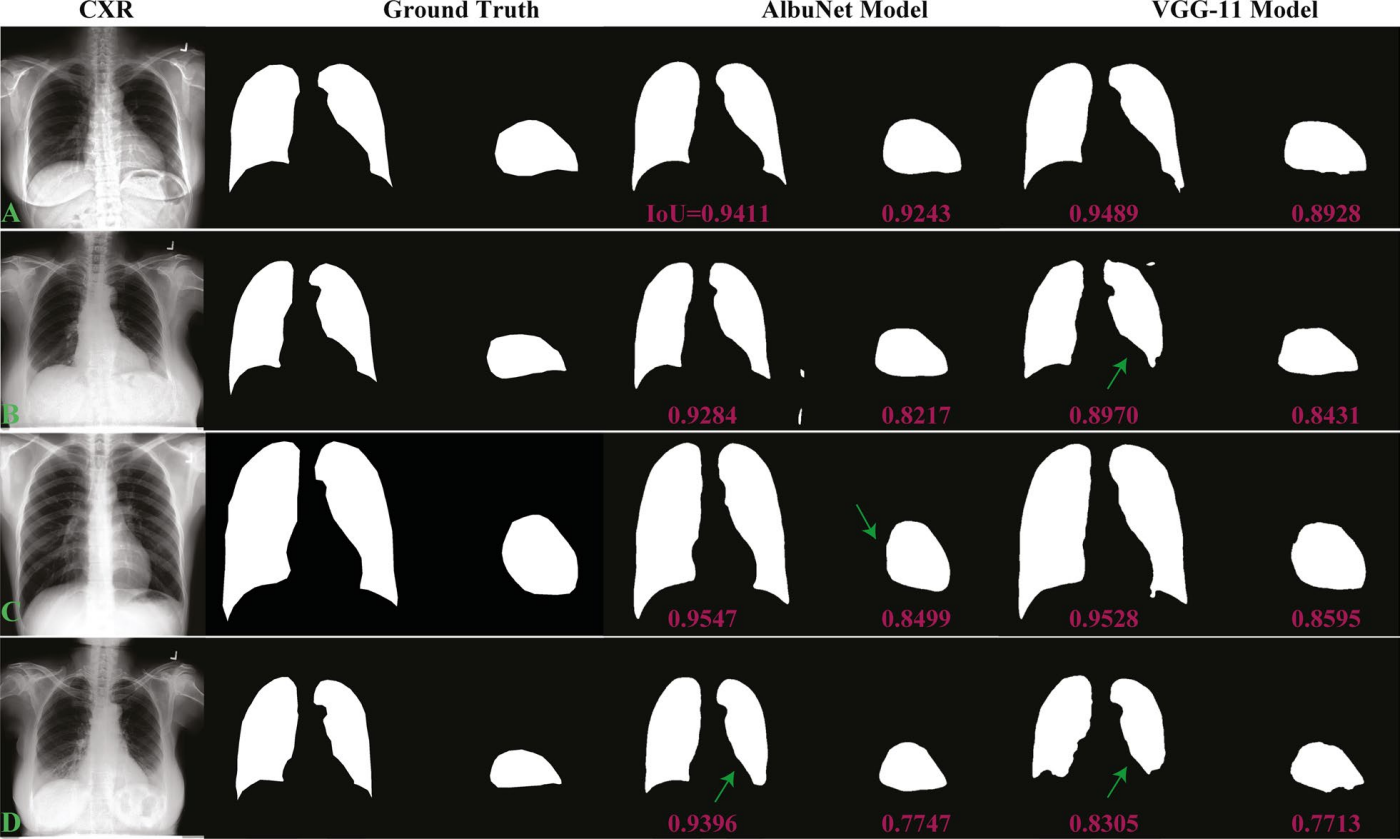
Segmentation of lung and heart region from AlbuNet and VGG-11 models of the same cases used in Fig. 1 with their Intersection over Union (IoU) values. The arrows point to the error of segmentations

There were no AI failure results from the AlbuNet+VGG-11 model applied on the evaluation dataset. Hence, only excellent and good grades were obtained (Table 5). Figure 1 demonstrated examples of the evaluation study with the excellent grade at the first three rows (Fig. 1a–i), and a good grade at the last row (Fig. 1j–l). Both Albunet and VGG-11 models obtained the excellent grade on the first row (Fig. 1a–c), and each model gave the excellent grade on the second (Fig. 1d–f) and third (Fig. 1 g–i) row, respectively. We observed that most failures on the VGG-11 model were due to underestimation of the internal diameter of the chest line (ID line) that caused the CTR values to be overestimated compared to manual operation (Fig. 1e, k). The AlbuNet model, on the other hand, underestimated the midline-to-right (MRD) or midline-to-left (MLD) heart diameter lines causing it to underestimate CTR values (Fig. 1g). Figure 4 demonstrates segmentation of the lung and heart regions from both models of the same cases used in Fig. 1 along with their Intersection over Union (IoU) values, these are the overlapping areas between the predicted and the ground-truth regions divided by the union of the two areas ranged from 0 (no overlap) to 1 (perfect overlap). The VGG-11 model seems to underestimate the lung region, especially around the shape edge region as compared to the AlbuNet model, while the heart contour from the AlbuNet model seems smoother, or smaller, than from the VGG-11 model (i.e., made minor underestimation of heart diameter).

**Table 5:** Grading of AI outcomes from combination of AlbuNet and VGG-11 models on evaluation dataset

User	Excellent grade	Good grade
User1	7926 (84.4%)	1460 (15.6%)
User2	7825 (83.3%)	1561 (16.7%)
User1 and User2	7299 (77.8%)	2087 (22.2%)

Intra- and inter-observer variations from the manual and AI-assisted methods using the AlbuNet+VGG-11 model on the evaluation dataset are presented in Table 6. Overall, the CV and bias of observer variations was lower than 1.6% and 0.32%, respectively. Furthermore, the model can achieve excellent grade results in about 78% of images (Table 5), which is quite comparable to the validation study (83%) with an average CTR measurement time of 1.07 ± 2.62 s per case, compared to 10.6 ± 1.5 s from manual operation in our previous study [9]. Thus, the combined AlbuNet+VGG-11 model could be clinically implemented to assist radiologists for CTR measurement because it can achieve the desired excellent-grade results, with low measurement variation and greater speed than manual operation.

**Table 6:** Bias, 95% CI, and coefficient of variation of intra- and inter-observer CTR measurements from Manual and AI-assisted methods using combination of AlbuNet and VGG-11 models on evaluation dataset

	Bias (95% CI) (%)	CV (%)
Intra-observer	− 0.10 (− 2.51 2.30)	0.88
Inter-observer	0.32 (− 3.97 4.61)	1.55

## Discussion

CTR measured from CXR images is a useful index to evaluate heart disease, especially cardiomegaly [1–4]. Manual measurement, however, is time consuming, especially if all CXR images need to be measured. DL tools can now provide reliable CTR measurement and may be implemented as an automated method [5–8, 10]. The tool can achieve measurement variation within the human-operation range, which is sufficient for research purposes, but in everyday clinical use, the measurements still require approval from radiologists. The DL tool in the clinical setting, therefore, has only been implemented as an AI-assisted method, rather than fully automated, with the aim to easing the burden of manual measurement.

The AI method has been successfully employed and validated to calculate CTR values [5–10]. Recently, our group demonstrated that an effective DL algorithm (AlbuNet model) could be implemented for automatic CTR measurement with average error on-par with manual expert measurement [10]. We investigated the performance and efficiency of our proposed DL models in the AI-assisted method as if it were employed for clinical use to measure CTR on all patients. We found that our combined AlbuNet+VGG-11 model could achieve measurement variation comparable to human operation, and obtain the desired excellent-grade results almost ten times faster than the manual operation. We also confirmed that the AlbuNet model gave the lowest CV of the single-mode models employed in the study. Its measurement variation was comparable to the inter-observer variation from the manual method (1.92% vs. 2.13%). The AlbuNet model, thus, is a preferred choice for CTR calculation for automated work such as research.

In the clinical setting, however, the measurement was implemented in a non-automated or AI-assisted method, which defined its success from the highest excellent grade results. From this definition, the VGG-16 model is preferable to AlbuNet because it provided more such results (68.9% vs. 57.1%), and its variation was still comparable to manual operation (2.12% vs. 2.13%). Due to improvements in the model architecture and training methodology, our new proposed model increased excellent grade results by more than 50% (40% vs. 68.9%) [9]. To further increase excellent-grade results, we investigated combined-model modes that were able to be implemented in the AI-assisted method, but not fully automated. We found that a combined-model mode could improve the frequency of excellent grade results with the best combination being the AlbuNet+VGG-11 model. We validated and evaluated the AlbuNet+VGG-11 model on validated and evaluated datasets and found that excellent grade results were comparable (82.7% and 77.8%), and higher than from the single-model mode. To the best of our knowledge, a combination-model has not been implemented before.

The AlbuNet+VGG-11 model can achieve high levels of excellent grade results due to the complimentary effect of both models. The AlbuNet model tends to underestimate CTR values compared to manual operation (i.e., correctly defined ID line but minor under-estimated MRD or MLD line due to smoother effect on heart contour compared to from the VGG-11 model). The VGG-11 model, on the other hand, tends to overestimate CTR values by underestimating the ID line (i.e., due to underestimation of lung segmentation around the sharp-edge region), but still gave reasonable estimation of MRD and MLD lines as demonstrated in Fig. 4. From the deep-learning perspective, since the cardiac silhouette is less defined than the thoracic boundary, segmentation models tend to make more errors on the estimation of cardiac boundaries. However, as described in our previous study [10], AlbuNet was shown to smooth out the contour and reduce outlier errors, with a tradeoff of slightly larger average errors. We postulated that this might be a result of AlbuNet’s residual connections. For a well-defined thoracic contour, smoothing is beneficial and tends to yield more accurate result, but for the blurry cardiac contour, smoothing can lead to an underestimated heart contour. Therefore, when AlbuNet results were minor underestimates, the user could select the complimentary VGG-11 result rather than making an adjustment, and vice versa. Thus, the combination of the two models increased the frequency of excellent grade results.

Furthermore, the AlbuNet+VGG-11 model also has lower measurement variation than manual operation (CV of 1.36% vs. 2.13%), which makes the method more acceptable for radiologists (i.e., most of the AI results were at reasonable values as compared to manual operation). There were, however, around 0.15% (data not shown) of cases that were extreme outliers (i.e., the AI results differed from manual operation more than the highest difference in the manual operation of two users), but these cases were uncommon and thought to be acceptable by our radiologists when using the AI-assisted method.

The performance of AlbuNet+VGG-11 model should reduce the workload of radiologists if the measurement is needed on all patients. In other words, the radiologist should be able to select the CTR results from the AI calculation in around 78% of cases, and the remainder will require only minor line adjustments. Implementation of this model could reduce operating time by almost five and ten-fold (1.07 ± 2.62 s vs. 2.2 ± 2.4 and 10.6 ± 1.5 s) as compared to our previous DL model [9] and manual operation, respectively. We plan to implement this model in our clinical setting to assist our radiologists with CTR measurement on all patients, and no longer measuring CTR only in suspicious cases. Furthermore, we plan to perform a pioneer study using the AlbuNet model to calculate CTR values on all CXR images of adult patients in our deposition (around one million images) to gain more insight into the CTR characteristics of our patients.

Our study has some limitations. We focused only on adult patients. Pediatric cases need to be further investigated and may require technical improvement before it can be implemented for clinical use. This study may be prone to biased performance due to the automated system implemented on a dataset from a single-site. A multi-site investigation is needed to test different CXR machines and patient ethnicities to further improve our understanding of the potential of this technology. To better explain the model, we also plan to investigate AI failures reported by users to gain more insight into the fairness and ethical use of our AI model.

## Conclusions

Our combined AlbuNet+VGG-11 model could be clinically implemented to assist radiologists with CTR measurement because it can achieve excellent-grade results in around 78% of images, has lower measurement variation, and is ten-fold faster to perform than manual operation. We conclude that our AI model can assist radiologists to perform CTR measurements on CXR images and thereby reduce the burden of measurement.

## Data Availability

The datasets generated during and/or analyzed in the current study are not publicly available due to patient data privacy concerns, but are available from the corresponding author on reasonable request.

## Abbreviations

AI: Artificial intelligence
CTR: Cardiothoracic ratio
CTR_diff_: Different CTR values between AI’s and manual result in percentage
CV: Coefficient of variation
CXR: Chest radiography
DL: Deep learning
ID: Internal diameter of chest at level of right hemi-diaphragm
MLD: Midline-to-left heart diameter
MRD: Midline-to-right heart diameter
PACS: Picture archiving communication system
